# Supplementary material for: Prevalence and Genotype-Phenotype Correlation of Lynch Syndrome in a Selected High-Risk Cohort from Qatar’s Population
Source: Genes (Basel). 2022 Nov 21;13(11):2176. doi: 10.3390/genes13112176 (PMC9690077; doi:10.3390/genes13112176)
Supplement: Supplementary file 1 [file genes-13-02176-s001.zip › Table S2.pdf]

Table S1. Genetic test results of Unaffected high-risk individuals with Lynch Syndrome.

Patient Code	Age/Gender	Nationality	Family History	Gene	Genetic test result					
					Nucleotide change	AA change	Variant type	Classification	Zygosity	Reported in LR/ Novel
U001	31/F	Qatari	-Known tribal variant in *PMS2* gene<br>-Paternal uncle: Prostate cancer dx.50's	*PMS2*	-	Deletion Exons 6-11	Large Deletion	P	Heterozygous	Novel
U002	54/F	Qatari	-Sister: endometrial cancer dx.40's (MMR loss on pathology)<br>-Father: liver	*MSH6*	c.2805dupT	p.Asp936Ter	Nonsense	P	Heterozygous	Reported

			cancer dx.70's<br>-Mother: liver cancer dx.65							
U003	61/F	Qatari	-Sister: breast cancer dx.30's - 40's<br>-Maternal aunt: stomach cancer dx.50's<br>-Paternal aunt: stomach cancer dx.>50	*PMS2*	IVS9-1G>T	-	Splice site	P	Heterozygous	Reported
U004	43/F	Qatari	-Maternal aunt: thyroid cancer dx.35 & breast cancer dx.30's	*EPCAM*	-	Deletion of entire EPCAM gene	Large deletion	P	Heterozygous	Novel

U005	66/F	Qatari	-Family hx is reportedly significant for young onset breast, colon and ovarian cancers	*EPCAM*	-	Deletion of Exons 1-9	Large deletion	P	Heterozygous	Reported
U006	43/F	Sudanese	-Mother: breast cancer and endometrial cancer dx.62	*PMS2*		Deletion of exons 13 and 14	Large deletion	P		Reported
U007	49/F	Qatari	No reported family hx of cancers.<br>-PMS2 gene variant found on her whole exome sequencing as part	*PMS2*	c.1376C>G	p.S459X	Nonsense	P	Heterozygous	Reported

			of the workup for hereditary glomerulosclerosis							
U008	53/F	Qatari	-Brother: colon cancer dx.45 -Two Daughters: CMMRD due to homozygous *PMS2* variant	*PMS2*	-	Deletions encompassing Exons 6- to11	Large deletion	P	Heterozygous	Novel
U09	22/M	Qatari	-Sister: CMMRD due to homozygous *PMS2* variant	*PMS2*	-	Deletions encompassing Exons 6- to11	Large deletion	P	Heterozygous	Novel
U010	31/M	Qatari	-Sister and brother: CMMRD due to	*PMS2*	-	Deletions encompassing	Large deleti	P	Heterozygous	Novel

			homozygous PMS2 variant			Exons 6- to11	on			
U011	45/M	Qatari	-Mother: colon ca. dx.60's (MMR loss)	*MSH6*	c.3946_3953delGGACATAG	p.Gly1316LysfsX6	Frame shift	P	Heterozygous	Reported

P: Pathogenic, LP: Likely Pathogenic, LR: Literature Review, LS: Lynch Syndrome, CMMRD: Constitutional Mismatch Repair Deficiency, dx.: diagnosis, P: Pathogenic.
