# Supplementary material for: Prevalence and Genotype-Phenotype Correlation of Lynch Syndrome in a Selected High-Risk Cohort from Qatar’s Population
Source: Genes (Basel). 2022 Nov 21;13(11):2176. doi: 10.3390/genes13112176 (PMC9690077; doi:10.3390/genes13112176)
Supplement: Supplementary file 1 [file genes-13-02176-s001.zip › Table S1.pdf]

Table S1. Clinical and genetic test results of affected individuals with Lynch Syndrome and CMMRD.

Patient Code	Age at diagnosis, Y/Sex	Nationality/Ethnicity	Diagnosis	immunochemistry				Genetic test results						
				MLH1	*MSH2*	*MSH6*	*PMS2*	Gene	Classification	Zygosity	Nucleotide change	AA change	Variant type	Reported/ Novel
C001	43/F	Filipino	LS	0	+	+	0	*MLH1*	P	Heterozygous	Exon 10 deletion	-	Large Deletion	Reported
C002	46/M	Nepalese	LS	0	+	+	0	*MLH1*	P	Heterozygous	IVS6+3A>G	-	Intronic	Reported
C003	53/M	British	LS	+	+	+	0	*MLH1*	P	Heterozygous	c.2252_2253delAA	p.Lys571SerfsX3	Frameshift	Reported
C004	45/M	Bangladeshi	LS	0	+	+	0	*MLH1*	P	Heterozygous	c.1633delA	p.Thr545ProfsX46	Frameshift	Novel
C005	48/M	Bangladeshi	LS	0	+	+	0	*MLH1*	LP	Heterozygous	IVS5-13A>G	-	Intronic	Reported
C006	35/F	Indian	LS	-	-	-	-	*MLH1*	LP	Heterozygous	c.1676T>C	p.Leu559Pro	Missense	Reported
C007	36/M	Egyptian	LS	0	+	0	0	*MLH1*	P	Heterozygous	c.1657delA	p.Thr553ProfsX38	Frameshift	Novel

C008	38/M	Egyptian	LS	0	+	+	0	*MLH1*	P	Heterozygous	c.1657delA	p.Thr553ProfsX38	Frameshift	Novel
C009	32/M	Sudanese	LS	0	+	+	0	*MLH1*	P	Heterozygous	Deletion Exon 6	-	Large Deletion	Reported
C0010	39/M	Indian	LS	+	0	0	+	*MSH2*	P	Heterozygous	IVS5+3A>T		Intronic	Reported
C0011	38/M	Filipino	LS	+	0	0	+	*MSH2*	P	Heterozygous	c.998G>A	p.Cys333Tyr	Missense	Reported
C0012	45/M	Sri Lankan	LS	+	0	0	+	*MSH2*	P	Heterozygous	c.301G>T	p.Glu101Ter	Nonsense	Reported
C0013	65/M	Palestinian	LS	+	0	0	+	*MSH2*	P	Heterozygous	c.754C>T	p.Gln252Ter	Nonsense	Reported
C0014	38/F	Egyptian	LS	+	0	0	+	*MSH2*	P	Heterozygous	c.229_230delAG	p.Ser77CysfsX4	Frameshift	Reported
C0015	59/M	Pakistani	LS	+	0	0	+	*MSH2*	P	Heterozygous	c.2090G>A	p.Cys697Tyr	Missense	Reported
C0016	60/F	Sudanese	LS	+	0	0	+	*MSH2*	P	Heterozygous	c.2446C>T	p.Gln816Ter	Nonsense	Reported
C001	56/M	Egyptian	LS	+	0	+	+	*MSH*	LP	Heterozyg	IVS11+2 T>C	-	Intronic	Novel

7								*2*		ous			
C0018	46/M	Nepalese	LS	0	0	+	+	*MSH2*	P	Heterozygous	c.1075_1076dupAG	p.Leu360AspfsX2	Frameshift	Reported
C0019	64/F	Qatari	LS	+	+	+	+	*MSH6*	P	Heterozygous	c.3475delT	p.Tyr1159ThrfsX25	Frameshift	Reported
C0020	-/F	Qatari	LS	-	-	-	-	*PMS2*	P	Heterozygous	c.1376C>G	p.Ser459Ter	Nonsense	Reported
C0021	23/F	Qatari	CMMRD	-	-	-	-	*PMS2*	P	Homozygous	deletions encompassing Exons 6- to11	-	Large Deletion	Novel
C0022	25/F	Qatari	CMMRD	-	-	-	-	*PMS2*	P	Homozygous	deletion encompassing Exons 6- to11	-	Large Deletion	Novel
C0023	28/F	Qatari	CMMRD	-	-	-	-	*PMS2*	P	Homozygous	deletions encompassing Exons 6- to11	-	Large Deletion	Novel

0: absence of expression; +: Presence of expression; -: not available, P: Pathogenic, LP: Likely Pathogenic, LR: Literature Review, LS: Lynch Syndrome, CMMRD: Constitutional Mismatch Repair Deficiency.
