# Supplementary figures and images for: Prevalence and Genotype-Phenotype Correlation of Lynch Syndrome in a Selected High-Risk Cohort from Qatar’s Population
Source: Genes (Basel). 2022 Nov 21;13(11):2176. doi: 10.3390/genes13112176 (PMC9690077; doi:10.3390/genes13112176)

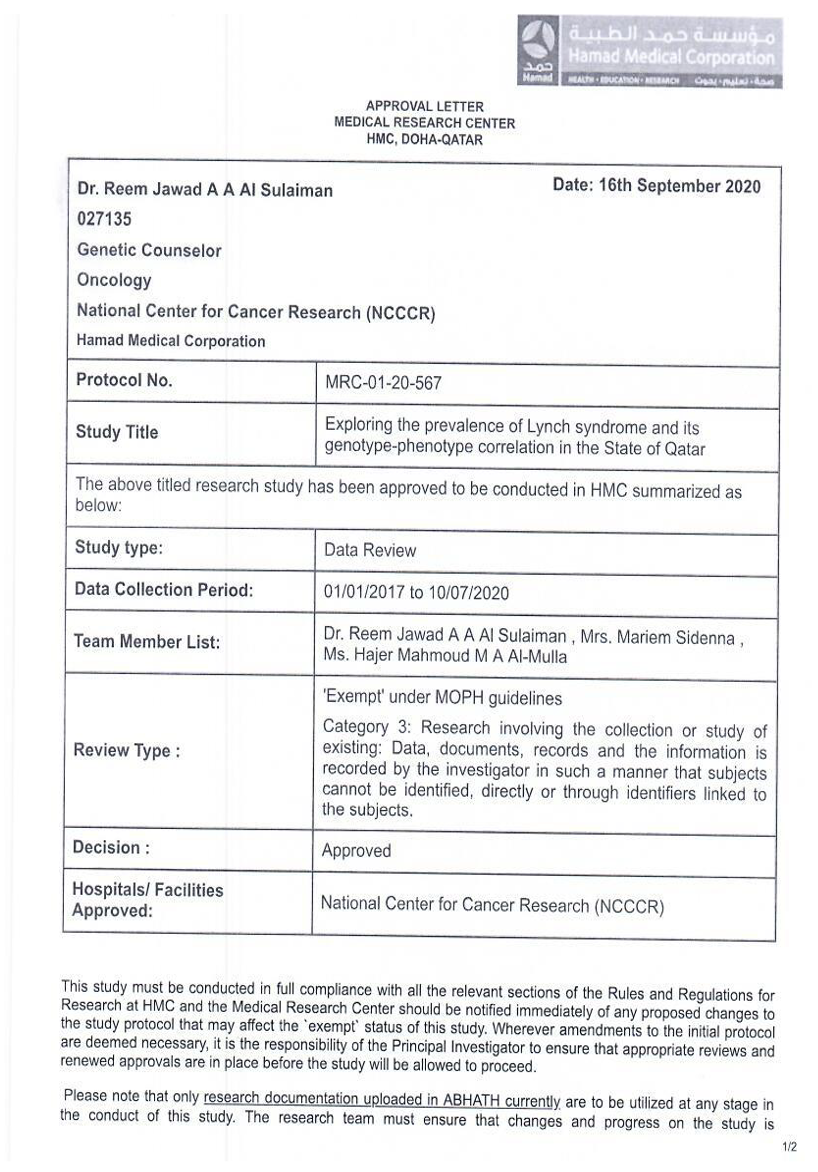

Supplement: Supplementary file 1 [file genes-13-02176-s001.zip › Figure S1.png]

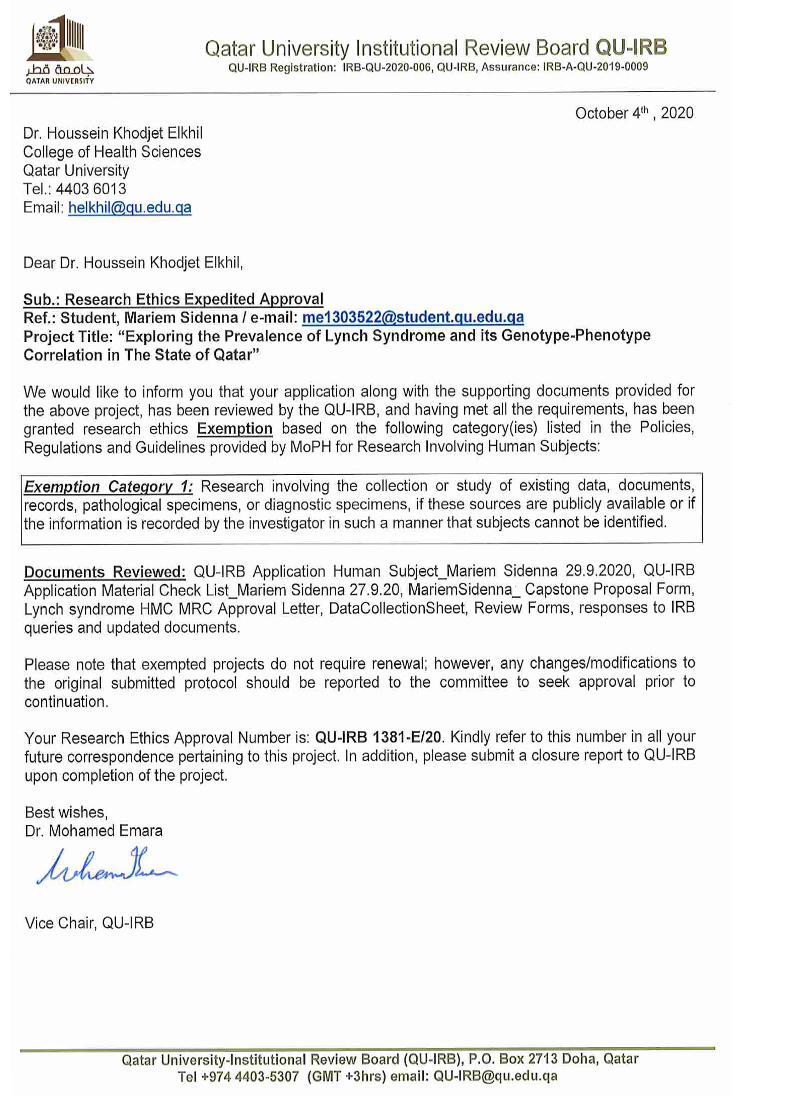

Supplement: Supplementary file 1 [file genes-13-02176-s001.zip › Figure S2.png]
